# Acoustic Biometric System Based on Preprocessing Techniques and Linear Support Vector Machines

**DOI:** 10.3390/s150614241

**Published:** 2015-06-17

**Authors:** Lara del Val, Alberto Izquierdo-Fuente, Juan J. Villacorta, Mariano Raboso

**Affiliations:** 1 Departamento de Ciencia de los Materiales e Ingeniería Metalúrgica, Expresión Gráfica de la Ingeniería, Ingeniería Cartográfica, Geodesia y Fotogrametría, Ingeniería Mecánica e Ingeniería de los Procesos de Fabricación, Área de Ingeniería Mecánica, Universidad de Valladolid, Paseo del Cauce 59, 47011 Valladolid, Spain; 2 Departamento de Teoría de la Señal y Comunicaciones e Ingeniería Telemática, Universidad de Valladolid, Paseo Belén 15, 47011 Valladolid, Spain; E-Mails: alberto.izquierdo@tel.uva.es (A.I.-F.); juavil@tel.uva.es (J.J.V.); 3 E.U. Informática, Universidad Pontificia de Salamanca, Calle Compañía 5, 37002 Salamanca, Spain; E-Mail: mrabosoma@upsa.es

**Keywords:** acoustic biometric system, acoustic images, preprocessing techniques, support vector machine

## Abstract

Drawing on the results of an acoustic biometric system based on a MSE classifier, a new biometric system has been implemented. This new system preprocesses acoustic images, extracts several parameters and finally classifies them, based on Support Vector Machine (SVM). The preprocessing techniques used are spatial filtering, segmentation—based on a Gaussian Mixture Model (GMM) to separate the person from the background, masking—to reduce the dimensions of images—and binarization—to reduce the size of each image. An analysis of classification error and a study of the sensitivity of the error *versus* the computational burden of each implemented algorithm are presented. This allows the selection of the most relevant algorithms, according to the benefits required by the system. A significant improvement of the biometric system has been achieved by reducing the classification error, the computational burden and the storage requirements.

## 1. Introduction

Biometric systems are based on the subject’s characteristics to allow his/her identification [1]. The main biometric systems use elements such as fingerprints, retina, face, voice, *etc*. to characterize people and then classify them for subsequent identification and validation. Each of these systems requires the use of specific sensors to obtain the desired characteristics of the subject. Video cameras are often used as sensors to identify subjects or property although a radar could also be used to obtain the shape of a subject through reflection [2,3]. There are accurate and reliable classification systems based on acoustic radars:

- Animal echolocation, developed by mammals such as bats, whales and dolphins through specific waveforms [4,5], or the identification of different types of flowers by other species [6].
- Acoustic signatures used in passive sonar systems [7,8], which analyze the signal received by a target in the time-frequency domain.

There is little literature on the use of an acoustic radar as a biometric system for human identification and an ultrasonic band rather than an audible frequency band is usually employed [9,10]. In previous works, the authors developed multisensor surveillance and tracking systems based on acoustic arrays and image sensors [11,12]. In another line of work, making the most of the adquired experience in acoustic arrays and image sensors, the authors developed a biometric identification system based on the acoustic images acquired with an electronically scanned array [13]. The system tries to discriminate subjects in terms of their acoustic image, directly related to the subject’s shape, height and geometrical characteristics. These characteristics are considered “soft biometrics” and they used to be used along with other “hard biometics” (e.g., fingerprints) in order to uniquely identify a person.

The system obtained acoustic images by scanning the subjects in four frequencies of the acoustic band and in four different positions, defining an acoustic profile that comprises all of these images. Subsequently, the acoustic profile was compared to previously stored profiles to identify the subject. In this first system, Mean Square Error (MSE) between two images of the same frequency and position is used to compare the acoustic profiles, defining a global error as the sum of the errors associated with each image of the profile. Using the Equal Error Rate (EER) as a quality indicator, this system obtained an EER value of 6.22%, such as other emerging biometric identification systems [14,15,16,17].

In a later work [18], the authors analyzed the contribution of each acoustic image—associated with a frequency and position—to the performance of the biometric system, finding that each image provides different degrees of information. Two main conclusions were obtained:

- Each set of images associated to certain frequency provides different information, improving the system performance, thus, the number of frequencies used should be increased.
- Information associated to certain subject positions only provides redundant information and does not improve the quality of the system, thus, the number of positions used should be decreased.

In a second stage of the analysis, a new global error function was proposed by weighting the MSE error of each image proportionately to the information that it provides. In this case, an EER value of 4% was obtained. The use of more efficient classification algorithms would provide an improvement in the classification error, which also represents an EER.

Since Support Vector Machines (SVMs) are algorithms that currently define Machine Learning [19], it was decided to work with them in the classification tasks. Furthermore, SVMs are the unique algorithms used in the classification capable of working with high-dimensional data, such as the case of the acoustic profiles used.

This paper presents an improved biometric system that uses a SVM algorithm for classification and identification of subject. Since high dimensionality of acoustic profiles exponentially increases the computational burden of SVM classifiers, preprocessing and feature extraction techniques have been designed and implemented to improve the classifier performance. This new system is based on the results obtained in previous studies [18].

In Section 2, SVM classification algorithms and associated training techniques are explained. Section 3 describes the biometric system, including acquisition, preprocessing and classification systems. In Section 4, an analysis of the results is done and finally, Section 5 presents the final conclusions.

## 2. Support Vector Machines

**Figure 1:**
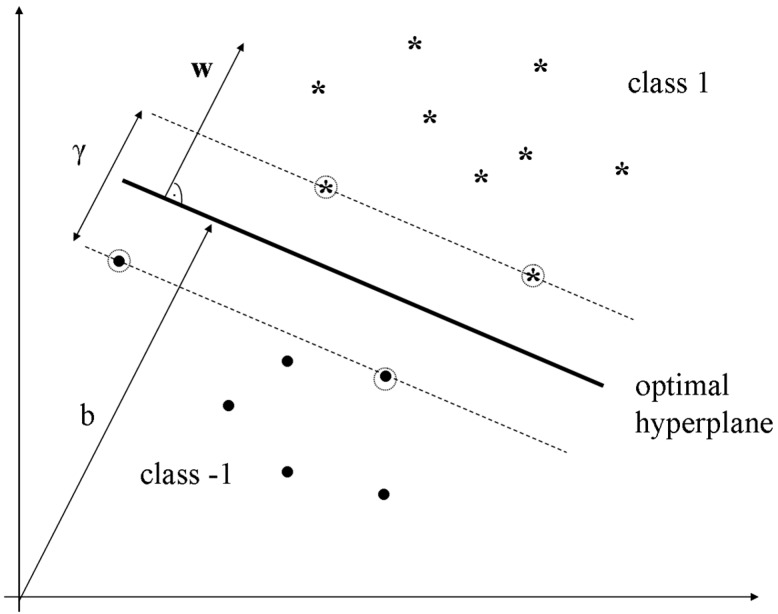
Hyperplane for binary classification.

SVMs carry out binary classification by constructing a hyperplane defined by the weight vector **w** and the bias term *b*, as shown in Figure 1, so samples of different classes will be divided by a separation, as wide as possible. Thereby, SVM algorithms are called maximum margin classifiers, being γ the margin of separation.

Based on a training set of *l* known samples formed by data vectors *x_i_* and the corresponding class labels *y_i_* to which they belong:

(1) $$\left(x_{1},y_{1}\right),\ldots ,\left(x_{l},y_{l}\right)\in R^{N}\times \left\{-1,1\right\}$$

Machine Learning algorithms obtain the hyperplane according to an optimization criterion, which must be validated subsequently.

In the validation phase, the class label of a new data vector *x* can be predicted by projecting **x** in the weight vector **w**:

f(x) = w · x + *b* (2)

The sign of this projection will reveal the predicted class label. Thus, new samples are mapped into the n-dimensional space and a class will be associated to them, depending on which side of the hyperplane has been mapped.

There are different possible hyperplanes that divide the data space into two subsets. Typically, the maximum margin criterion is used as an appropriate optimization criterion to obtain the hyperplane with the greater margin of separation *γ* (see Figure 1). Only the vectors (or samples) positioned on the margin—which are called support vectors and that in Figure 1 are surrounded by a circle—are necessary to describe this hyperplane.

For a canonical representation of the hyperplane, the constraints *y_i_* (**w**·**x**_i_+*b*) ≥ 1 must be met to find the margin γ = 2/||**w**||. The maximization of margin γ is equivalent to the minimization of (1/2) ||**w**||^2^, subject to the same restrictions.

Violation of restrictions involves the introduction of the variable ξ_i_, giving rise to the so-called problem of soft-margin SVM optimization:

(3) $$\begin{array}{ccccc} \min & \frac{1}{2}{\left\|w\right\|}^{2}+C\sum_{i}\xi_{i} & s.t. & y_{i}(wx_{i}+b)\geq 1-\xi_{i}, & \xi_{i}>0\forall i \end{array}$$

C is the regularization parameter, so that higher values of C correspond to stronger violations penalties.

In order to resolve the problem showed in Equation (3), it is rewritten in terms of positive Lagrange multipliers α_i_. In this way, it is required to maximize the following expression:

(4) $$L_{D}=\sum_{i=1}^{l}\alpha_{i}-\frac{1}{2}\sum_{i,j=1}^{l}\alpha_{i}\alpha_{j}y_{i}y_{j}\left(x_{i}\cdot x_{j}\right)$$

subject to restrictions 0 ≤ α_i_ ≤ C and ∑_i_ α_i_y_i_ = 0, given the relation:

(5) $$w=\sum_{i}^{N_{s}}y_{i}\alpha_{i}x_{i}$$

where Ns denotes the number of resulting support vectors. The discriminant function on which the SVM optimization is based is obtained by substituting **w** in Equation (2):

(6) $$f\left(x\right)=\sum_{i}^{N_{s}}y_{i}\alpha_{i}\left(x\cdot x_{i}\right)+b$$

Essentially, a SVM is a two-class classifier but, in practice, it is very common to find problems associated with *K* > 2 classes. In these cases, a multiclass classifier is needed. There are several methods that combine multiple two-class SVM to obtain a multiclass classifier. The most widespread methods are the one-*versus*-all and the one-*versus*-one [19].

Training and Validation

**Figure 2:**
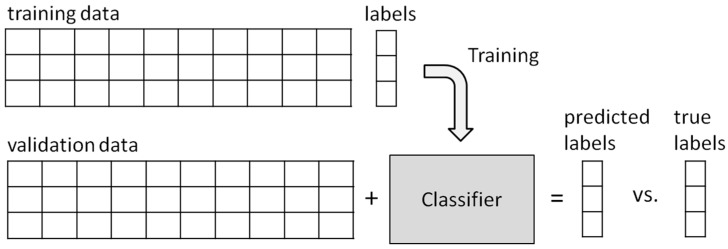
Classifier training.

The classifier learns from a training set—samples whose class labels are known—and defines a hyperplane. Then, this hyperplane is used to classify the samples from the validation set—whose classes are unknown. After that, the associated classes are compared with their corresponding classes and the error rate of the classifier is assessed, as shown in Figure 2.

The number of available samples is finite (*N* samples) and should be divided among the training set and the validation set. In this work, the classification algorithm is trained, using the two most common training methods:

- Leave-One-Out (LOO)
- Cross Validation (CV)

In LOO [20], training is carried out using *N* − 1 samples, and validation is performed using the sample which has been excluded. Errors are taken into account when the classification is wrong. This process is repeated *N* times, each time excluding a different sample. The total number of errors gives an estimation of the classification error rate.

**Figure 3:**
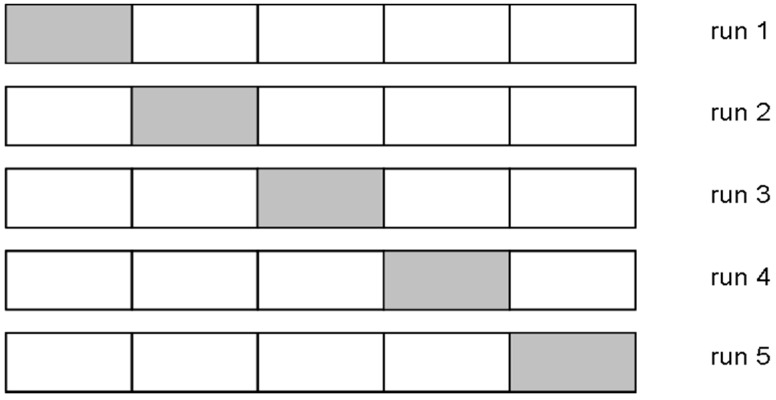
5-fold Cross Validation.

On the other hand, the CV method involves taking the available data samples and dividing them into *S* groups (named folds) [21]. *S*-1 folds are used to train the model, and the remaining fold is used to validate. This procedure is repeated *S* times, taking a different fold each time to validate the model. Finally, the classification error rate is the average of the errors that have been obtained in each of the *S* runs. An example of a 5-fold cross-validation (*S* = 5) is shown in Figure 3, where the fold used to validate is highlighted.

## 3. System Description

**Figure 4:**
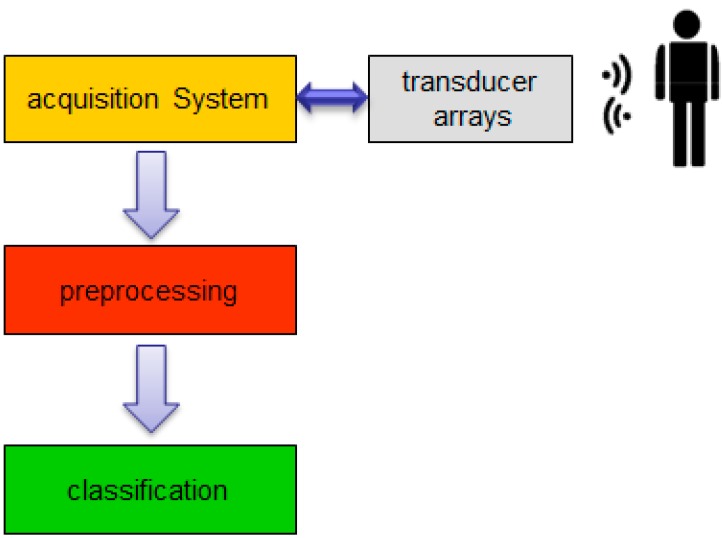
Functional description block diagram.

Based on basic radar/sonar principles [22,23], an acoustic detection and ranging system for biometric identification was proposed [24], according to the block diagram in Figure 4.

This system performs four main tasks: (i) subject scanning; (ii) acoustic images acquisition; (iii) images preprocessing and (iv) subject identification, based on classification algorithms.

### 3.1. Acquisition System

**Figure 5:**
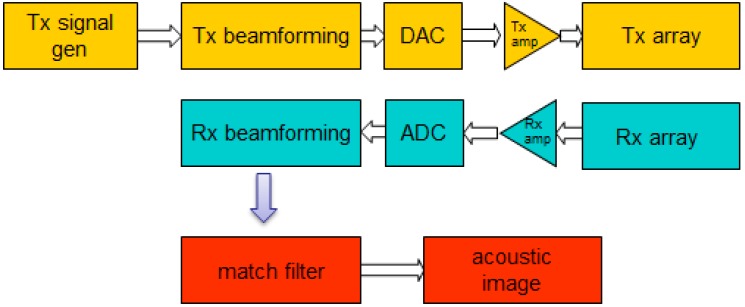
Acquisition system block diagram.

The subject is electronically scanned in the azimuth coordinates using two linear arrays. For each steering angle the system performs: (i) transmission beamforming; (ii) reception beamforming and (iii) match filtering in the range coordinate. After processing all the steering angles, a two-dimensional matrix is formed and stored, representing the acoustic image. Figure 5 shows the block diagram for the acquisition system.

**Figure 6:**
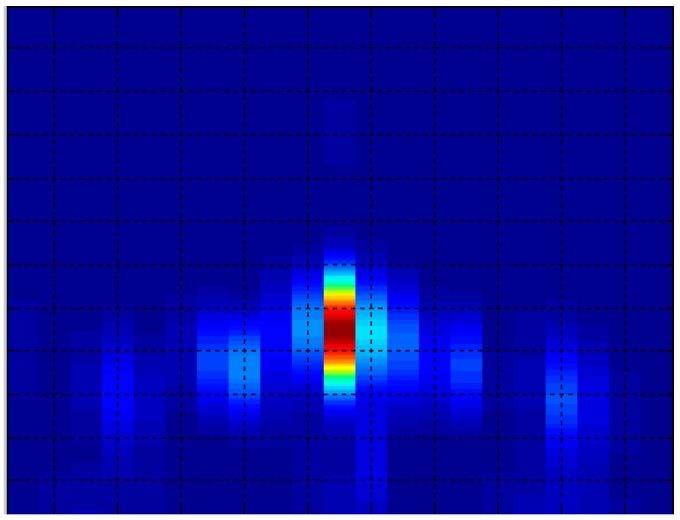
Acoustic image example.

Figure 6 shows an example of an acoustic image, considering that the x axis represents the azimuth angle and the y axis, the range.

Based on the conclusions of previous works [18], a new system that employs P = 3 spatial positions and F = 9 frequencies is defined. This system generates P_i_ acoustic profiles, associated to subject i and formed by P·F = 27 images. 

The selected positions for the subject under analysis are: front view with arms outstretched (*p_1_*), back view (*p_2_*) and side view (*p_3_*). The nine frequencies are 500 Hz-spaced, from 8 kHz (*f_1_*) to 12 kHz (*f_9_*). The number of beams used for each frequency is shown in Table 1. 

**Table 1:** Number of beams *vs.* frequency.

f_1_	f_2_	f_3_	f_4_	f_5_	f_6_	f_7_	f_8_	f_9_
13	15	15	17	17	17	19	19	21

### 3.2. Preprocessing and Parametrization Techniques

With the purpose of reducing the dimension of the acoustic profiles, eliminating redundant or non-significant information and thus reducing the associated computational burden, several preprocessing and parametrization techniques on acoustic images have been evaluated. Among the preprocessing techniques, the following processes are implemented:

- spatial filtering
- segmentation using Gaussian Mixture Models algorithms
- masking
- binarization

On the other hand, a reduced set of parameters was extracted from the acoustic images in order to characterize them. Two families of algorithms were analyzed:

- line-based image coding
- geometric feature extraction

**Figure 7:**
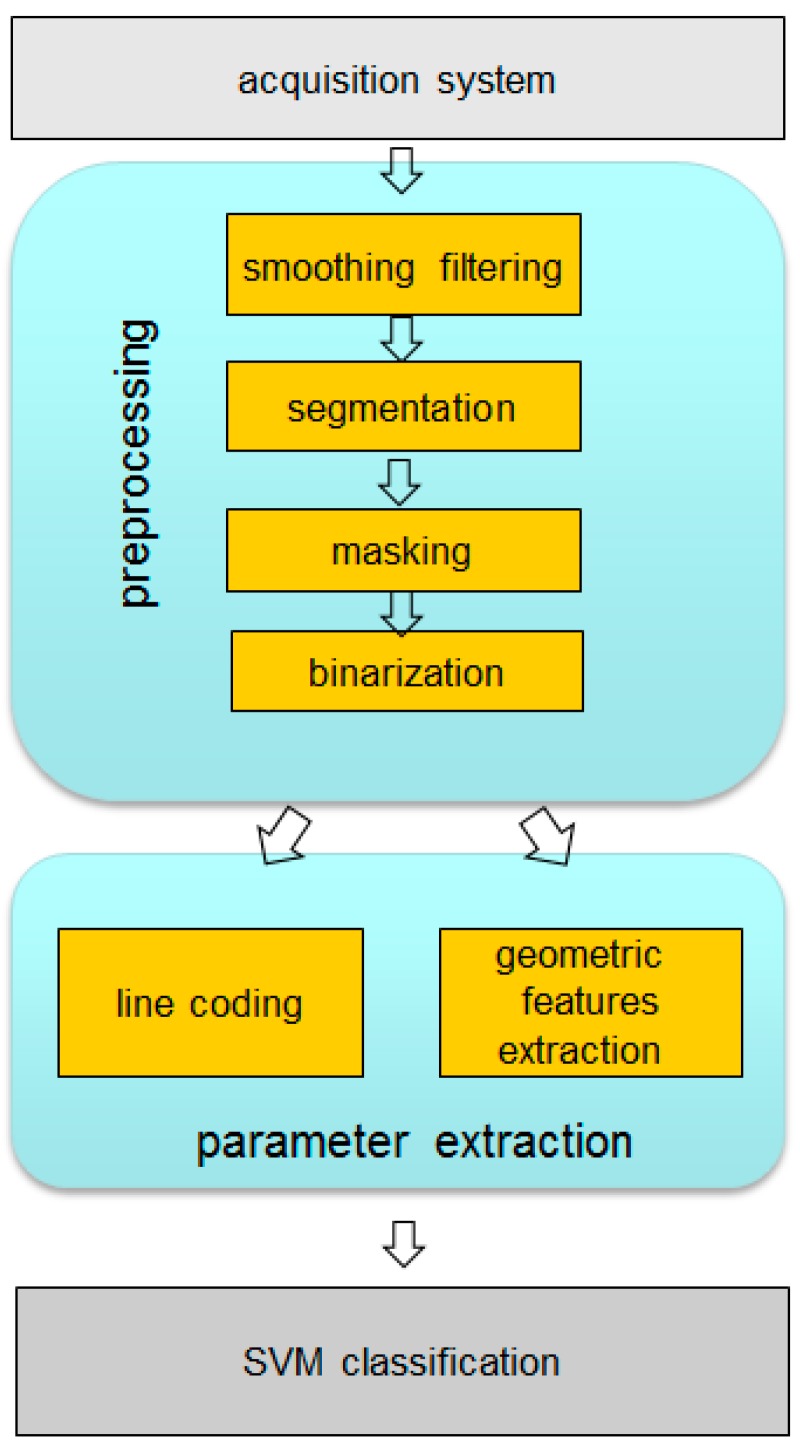
Preprocessing and parametrization techniques.

Figure 7 shows the processing scheme.

First, a spatial filter was implemented to smooth images, in order to reduce multimodalities of torso echoes and improve the segmentation process [10]. Then, a segmentation algorithm was used to differentiate pixels associated to the object from the pixels associated to the background.

The Expectation-Maximization (EM) algorithm is used to adjust a Gaussian Mixture Model (GMM) formed by two Gaussians, associated with foreground and background, respectively. The pixels associated with the background are zeroed. 

The dimensions of the images N × M—where N is the number of rows (dimension in range) and M is the number of columns (dimension in azimuth)—are detailed for each frequency and position in Table 2. 

**Table 2:** Image sizes.

N × M	f_1_	f_2_	f_3_	f_4_	f_5_	f_6_	f_7_	f_8_	f_9_
**p_1_, p_2_, p_3_**	245 × 13	245 × 15	245 × 15	245 × 17	245 × 17	245 × 17	245 × 19	245 × 19	245 × 21

The profiles formed by the acoustic images are stored to be processed and that is why the size of each pixel of the images has to be defined. The final size of the profiles gives the required storage space, and is related with the computational burden associated to the system. In this case, the value of each pixel is stored in memory using B = 32 bits.

Using masking techniques, the size of the images is reduced by adjusting them to the area that the subjects take up on the image. A statistical analysis of the acquired images was performed to determine the common area for each position and frequency. The sizes of the images obtained by this technique for each frequency and position are detailed in Table 3.

**Table 3:** Masked image sizes.

N × M	f_1_	f_2_	f_3_	f_4_	f_5_	f_6_	f_7_	f_8_	f_9_
**p_1_**	145 × 13	145 × 15	145 × 15	145 × 17	145 × 17	145 × 17	145 × 19	145 × 19	145 × 21
**p_2_**	155 × 11	155 × 11	155 × 11	155 × 11	155 × 11	155 × 11	155 × 11	155 × 11	155 × 11
**p_3_**	171 × 9	171 × 9	171 × 9	171 × 9	171 × 9	171 × 9	171 × 9	171 × 9	171 × 9

**Figure 8:**
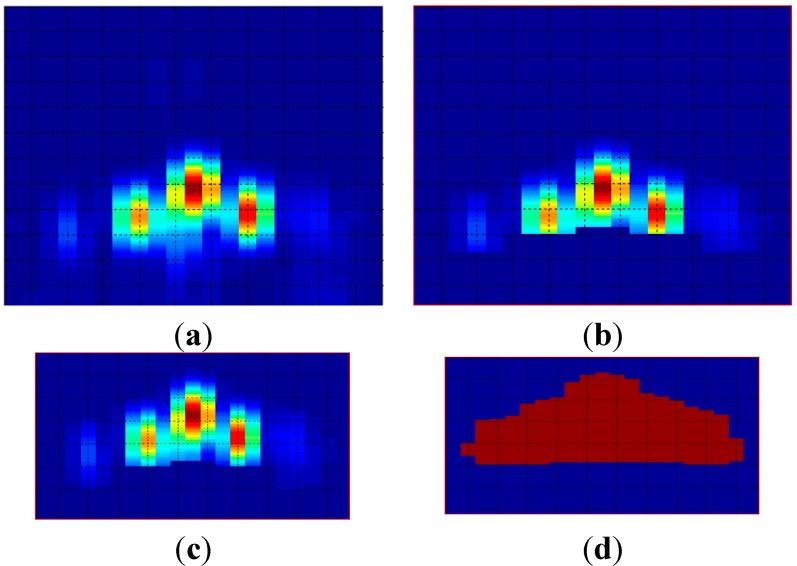
Pre-processed images: (**a**) original; (**b**) segmented; (**c**) masked; (**d**) binarized.

Finally, the value of the pixels—encoded with 32 bits—is reduced to 1 bit by image binarization. This significantly reduces the storage space required. This operation is equivalent to associate a unit value to the foreground pixels. Figure 8 shows an example of the use of the preprocessing techniques, showing an original Figure 8a, a segmented Figure 8b, a masked Figure 8c and a binarized Figure 8d image.

**Table 4:** Image sizes using Row-based Image Coding.

L	f_1_	f_2_	f_3_	f_4_	f_5_	f_6_	f_7_	f_8_	f_9_
**p_1_**	145	145	145	145	145	145	145	145	145
**p_2_**	155	155	155	155	155	155	155	155	155
**p_3_**	171	171	171	171	171	171	171	171	171

Starting from the binarized images, two feature extraction techniques were applied, significantly reducing the size of the acoustic images. First, Line-based Image Coding algorithms were analyzed. The images are broken down into a set of lines that can be rows or columns. For each line, the number of pixels with unit value is encoded. In this way, the size of each image is significantly reduced, from a N·M size to a L size, where L is N or M, as encoding is performed by rows or columns, respectively. The value of each parameter is stored in the memory using B = 8 bits. In row coding, sizes of the images obtained at each position and frequency are shown in Table 4.

In column coding, sizes of the images obtained for each frequency position are detailed in Table 5.

**Table 5:** Image sizes using Column-based Image Coding.

L	f_1_	f_2_	f_3_	f_4_	f_5_	f_6_	f_7_	f_8_	f_9_
**p_1_**	13	15	15	17	17	17	19	19	121
**p_2_**	11	11	11	11	11	11	11	11	11
**p_3_**	9	9	9	9	9	9	9	9	9

**Figure 9:**
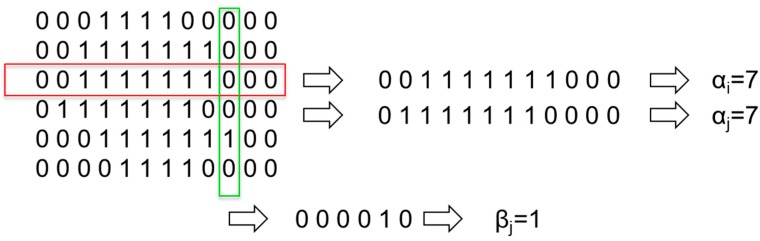
Line-based Image Coding.

As an example, Figure 9 represents Line-based Image Coding using rows and columns of an acoustic image of size 6 × 12.

**Figure 10:**
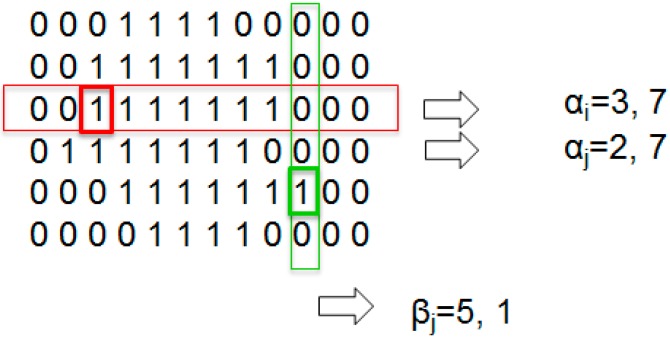
Line-based Image Coding with position.

In Figure 9, it can be observed that rows 3 and 4 gave an identical encoding, although they are different. To improve the information of each line and avoid ambiguous encodings, a second parameter that stores the starting position of the first nonzero pixel per line is added. With this improvement, the image dimension is doubled. As an example, Figure 10 represents the new encoding methods for the previous image.

Secondly, geometric feature extraction algorithms were analyzed. They show the following properties of images:

- Area : A
- Centroid: (c_x_, c_y_)
- Perimeter: P

**Figure 11:**
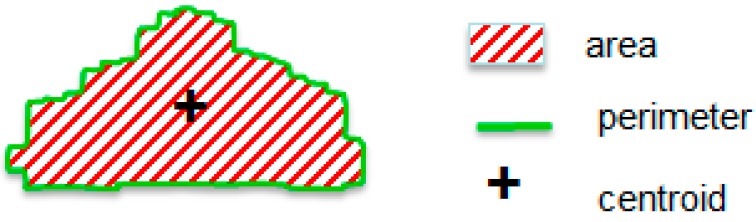
Geometric feature extraction.

In this case, one parameter for area and perimeter, and two parameters for centroid are extracted from each image. The value of each parameter is stored in the memory using B = 32 bits. Figure 11 shows the geometric features extracted from Figure 8d.

### 3.3. Classification

In this work, tests based on linear SVM algorithms were performed. A linear SVM was used because, if the number of features is large, one may not need to map data of a higher dimensional space and using the linear kernel is good enough [25]. Besides, although the dimension of the acoustic images is reduced with the preprocessing techniques, it is still too high in order to be used in SVMs based on a Gaussian kernel, involving an increment of the processing time and the computational burden, without an improvement in the classification error rate.

It was implemented with Matlab, specifically using the LIBSVM library, which allows multiclass SVM classification according to the *one-versus-one* algorithm [26]. The methods LOO and CV, with 10, 5 and 4 folds were used for algorithm training.

In the linear SVM, the regularization parameter C was set to 5000, since a usual practice is to assign C to the range of output values of the SVM algorithm [27], *i.e.*, the maximum number of possible errors, which coincides with the total number of samples.

## 4. Analysis of Results

### 4.1. Scenario Definition 

This study assumes that the system is used as an access control to enter in a laboratory, where only five subjects have authorized access. The SVM algorithm must be able to classify the subjects who try to access the laboratory in six different classes:

- a class for each of the five authorized subjects
- a class associated to all other people, considered as intruders.

To evaluate the performance of the SVM classification algorithm in acoustic biometric system, 5000 profiles were used. They are divided into six classes according to the following distribution:

- 500 acoustic profiles for each of the five authorized people.
- 2500 acoustic profiles for 25 intruders.

In order to have a population sample as general as possible, the subjects whose acoustic images were used have different morphological characteristics, as shown in Table 6. In this case, unlike previous tests [13,18], acoustic images of each subject were obtained on different days and with them wearing different clothes. In this way, the system will be able to classify the subjects according to who they actually are, without clothes being a distinctive factor.

**Table 6:** Morphological features.

Id	# Signatures	Gender	Constitution	Height
**Authorized**				
00	500	male	thin	average
01	500	female	normal	average
02	500	female	normal	small
03	500	male	strong	tall
04	500	male	very strong	tall
**Intruders**				
05-29	125	male	strong	average
	150	female	thin	average
	150	male	thin	average
	300	female	normal	average
	100	male	very strong	tall
	125	male	normal	average
	125	male	strong	tall
	100	female	normal	small
	125	female	strong	small
	125	female	strong	tall
	325	male	strong	average
	350	male	thin	small
	400	female	thin	small

**Figure 12:**
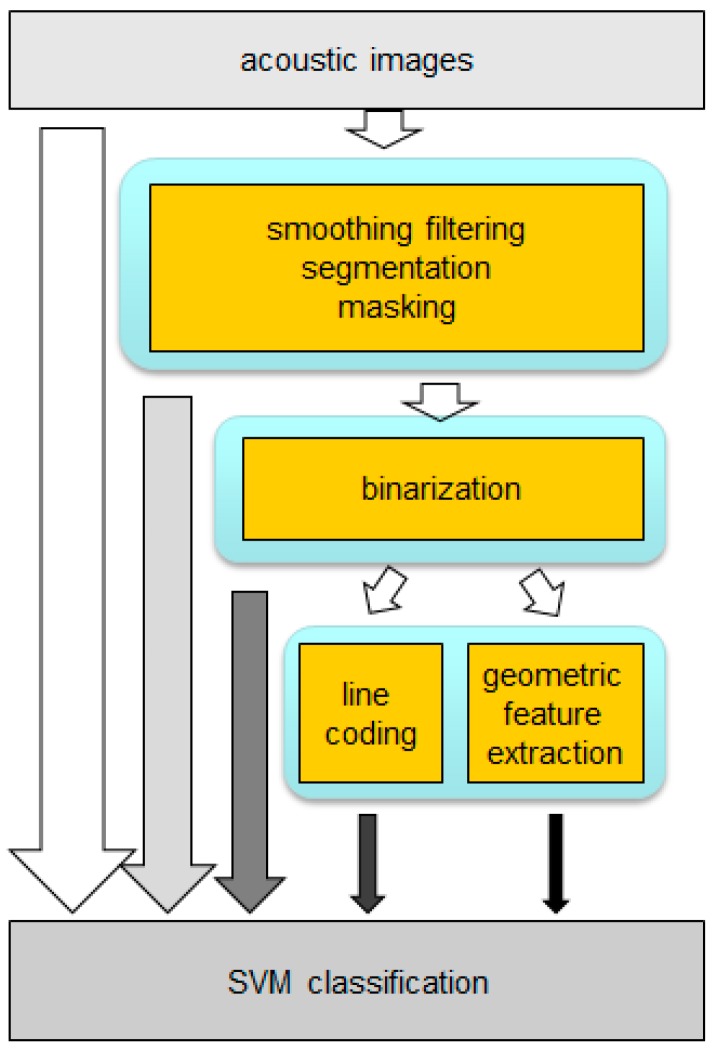
Experiments.

Based on this scenario, a set of experiments were conducted to analyze the performance of the proposed classification algorithm using different acoustic profiles. Figure 12 shows experiments that were carried out in this study. The system was tested with raw, preprocessed, binarized, line-based encoded and geometric features extracted profiles.

### 4.2. Raw Profiles

These acoustic profiles use raw images, without preprocessing. Each of the profiles has a size in the order of 3.5 × 10^6^; this value is obtained from the following Equation (7):

(7) $${\text{size}}_{\text{profile}}=B\cdot \overset{P}{\underset{i=1}{\sum }}N_{i}\cdot \overset{F}{\underset{j=1}{\sum }}M_{i,j}$$

where B is the number of bits used to store the value of each pixel of the acoustic images, P is the number of positions used in the system, N_i_ is the number of rows for each position, F is the number of frequencies and M_i,j_ is the number of columns for each position and frequency. The specific values of these variables are shown in Section 3.2.

In this test, an average classification error rate of 0.46% with a standard deviation of 0.120 was obtained. Comparing the error rate obtained using SVMs, which represents an Error Equal Rate, with the EER obtained with the classifier based on mean squared error (MSE), the classification error rate was reduced significantly, *i.e.*, from 4% [18] to 0.46%.

### 4.3. Preprocessed Profiles

In this case, raw profiles were first filtered, then segmented via GMM and, finally, masked, as explained in Section 3.2. Now, each preprocessed profile has a storage size in the order of 1.6 × 10^6^. This value was obtained using Equation (7).

A mean error classification rate of 0.46% with a standard deviation of 0.121 was obtained. Comparing these results with those obtained using raw profiles, it can be observed that when the profile size is reduced-equivalent to reducing computational burden-the error rate does not change. This shows that eliminated data does not provide relevant information to the classification task.

After that, the preprocessed profiles were binarized. Each profile has now a size in the order of 4 × 10^5^. In this case, an error rate of 0.75% with a standard deviation of 0.255 was obtained. If these results are compared with those obtained using preprocessed profiles without binarization, it can be observed that size reduction slightly increased the classification error rate, however, at a lower ratio than the reduction in size. 

This shows that the most relevant data of the profiles is related to the shape of the subjects, not to the specific value of their pixels. This is why working with binarized profiles is the next step to achieve size reduction. Table 7 summarizes the results obtained in these tests, corresponding to the classification based on raw, preprocessed and binarized acoustic profiles.

**Table 7:** Classification error rates for raw, preprocessed and binarized acoustic profiles.

Acoustic Profile	Error Rate	σ (Error Rate)
**Raw**	0.46%	0.120
**Preprocessed**	0.46%	0.121
**Binarized**	0.75%	0.255

### 4.4. Parameter Extraction

#### Line-Based Image Coding

First, line-based image coding was done using the length of rows or columns of the acoustic images. Then, the algorithm was improved, including the initial position of rows and columns. The size of the acoustic profiles obtained through this line coding is calculated using Equation (8):

(8) $${\text{size}}_{\text{profile}}=B\cdot K\cdot \overset{P}{\underset{i=1}{\sum }}\overset{F}{\underset{j=1}{\sum }}L_{i,j}$$

where B is the number of bits used to store the value of each parameter, K is the number of parameters used to encode each line—whose possible values are shown in Table 8—P and F are the number of positions and frequencies, respectively, and L_i,j_ is the number of lines, *i.e.*, rows, columns or the sum of both. The specific values of these variables are shown in Section 3.2.

**Table 8:** Number of parameters per line.

Line-Based Image Coding	K
**Based on Line Length**	1
**Based on Line Length and Position**	2

In the first case, profiles have a size in the order of 3 × 10^4^, 2.6 × 10^3^ and 3.6 × 10^4^, when row, columns and both rows and columns are encoded. The results obtained in these tests are shown in Table 9. Line coding based on length reduces considerably the size of the acoustic profile, although classification error rate increases. On the other hand, despite error rate getting worse, its values are still acceptably low. The lowest error rate value is obtained using both row and column coding, with a mean value of 1.43% and a standard deviation of 0.390.

**Table 9:** Classification error rates for line coding based on line length.

Line Coding	Error Rate	σ (Error Rate)
**Row**	1.93%	0.498
**Column**	1.97%	0.546
**Row + Column**	1.43%	0.390

In the second case, profiles are twice as large as the ones in the first case, since the initial position of the line is also encoded. These sizes are in the order of 6.8 × 10^4^, 5.3 × 10^3^ and 7.3 × 10^4^, if rows, columns or both rows and columns are encoded. In this case, although the profile dimension increases, the classification error rate decreases. If both row and columns are encoded according to length and position, an error rate of 0.47% is obtained. This error rate value is similar to the one obtained when using raw profiles. The obtained results are shown in Table 10. 

**Table 10:** Classification error rates for line coding based on length and position.

Line Coding	Error Rate	σ(Error Rate)
**Row + Position**	1.47%	0.407
**Column + Position**	1.86%	0.391
**Row + Column + Position**	0.46%	0.061

### 4.5. Geometric Feature Extraction

For this set of tests, the size of the profiles obtained by extracting geometric features of the acoustic images is calculated using Equation (9):

(9) $${\text{size}}_{\text{profile}}=B\cdot K\cdot P\cdot F$$

Where B is the number of bits needed to store the value of each parameter, K is the number of extracted features; P and F are the number of positions and frequencies, respectively.

The sizes of these acoustic profiles are in the order of 8.6 × 10^2^, if area or perimeter are used as geometric feature, and 1.7 × 10^3^, if centroid is employed. It can be observed that using geometric features reduces profile size, but the classification error rate increases excessively, as it is shown in Table 11. The obtained error rates are between 11% and 15%. These values are considerably higher than the reference error rate of 4% [18]. 

**Table 11:** Classification error rates using geometric features.

Geometric Parameters	Error Rate	σ(Error Rate)
**Area**	11.07%	0.297
**Centroid**	12.04%	0.310
**Perimeter**	15.04%	0.325
**Area + Centroid + Perimeter**	6.86%	0.430

Aiming to reduce error rate, the employed features were joined to form new profiles. In this case, although the profile size increases, the obtained error rate is reduced to 6.86%, as Table 11 shows. This value of the error rate is above the reference one.

### 4.6. Results Discussion

**Figure 13:**
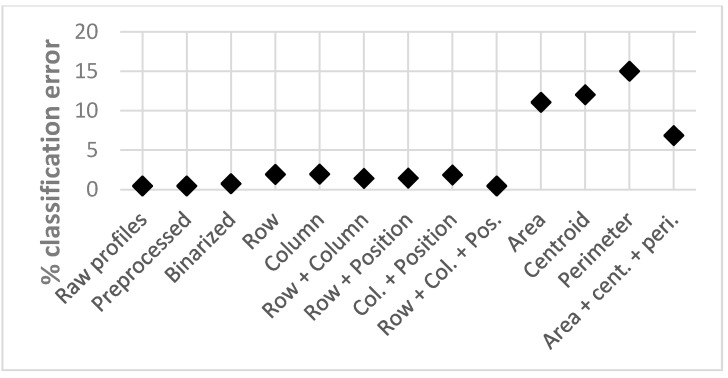
Classification error rates.

**Figure 14:**
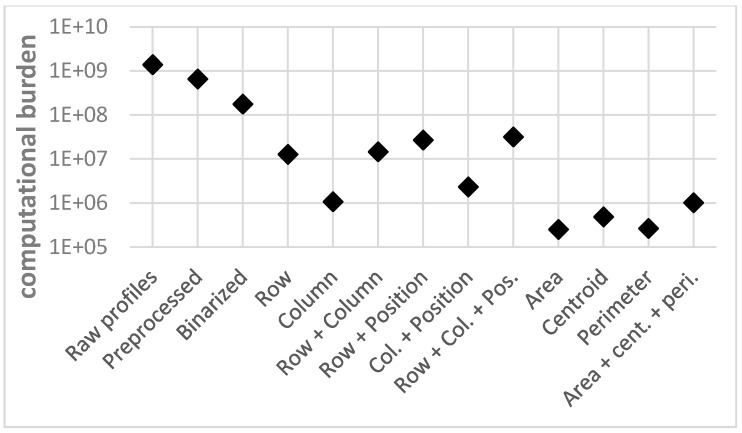
Computational burden.

Figure 13 shows the classification error rate obtained for each test, and Figure 14 shows the corresponding computational burden. This computational burden is calculated as the product of the number of support vectors, employed by the SVM for the classification, and their size.

In order to analyze these parameters and their relationship, classification error and computational burden sensitivities were defined as follows:

- Classification error sensitivity
  (10) $${\text{S}}_{\text{e}}=\frac{\left(pre\right)processed\_error}{raw\_error}$$
  with *raw*_*error* as the error using raw profiles.
- Computational burden sensitivity
  (11) $${\text{S}}_{\text{b}}=\frac{\left(pre\right)processed\_burden}{raw\_burden}$$
  with *raw_burden* as the burden using raw profiles.

Error sensitivity shows how the error rate increases due to profile size reduction. In the same way, burden sensitivity shows how burden decreases due to profile size reduction. Given that S_b_ values are always lower than 1, 1/S_b_ has been analyzed in order to compare both sensitivities in a similar way. Sensitivity values are shown in Table 12.

**Table 12:** Classification error and computational burden sensitivities.

	S_e_	1/S_b_
**Raw Profiles**	1.00	1.00
**Preprocessed**	1.00	2.13
**Binarized**	1.62	7.85
**Row**	4.17	109.72
**Column**	4.25	1297.28
**Row + Column**	3.09	95.02
**Row + Position**	3.17	51.43
**Col. + Position**	4.02	599.31
**Row + Col. + Pos.**	1.00	43.90
**Area**	23.94	5581.96
**Centroid**	26.02	2883.10
**Perimeter**	32.51	5242.42
**Area + Cent. + Peri.**	14.83	1382.36

**Figure 15:**
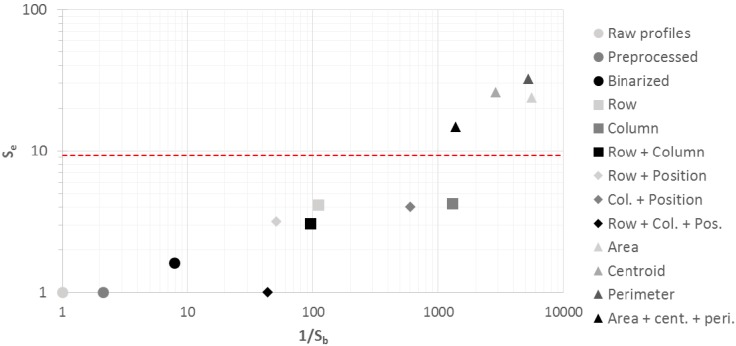
Error increment vs. burden reduction.

Figure 15 shows 1/S_b_ versus S_e_ in order to evaluate the relationship between error increment and burden reduction.

The dashed line in Figure 15 represents a reference S_e_ value of 9.30, which corresponds to the reference error rate, 4%, obtained in previous works. Thus, those algorithms—whose error variation sensitivity is higher than this reference value—should not be taken into account. The use of geometric features of the acoustic images reduces computational burden by a factor close to 1400, but the classification error increases 14%, so they must be discarded. 

The optimal working area, placed in the lower right part of Figure 15, corresponds to a high reduction of the computational burden—high 1/S_b_ values—and a low increment of the error classification rate—low S_e_ values. However, computational burden reduction involves a reduction of the amount of data that represents the acoustic profile, which brings about the possible elimination of relevant information for the biometric classification and the increment of the classification error.

On the other hand, those algorithms which eliminate non-relevant information of the acoustic profiles reduce the computational burden without an increment of the error classification rate. The preprocessed algorithm and the line coding algorithm based on length and position of rows and columns present the same error classification rate as the algorithm which uses raw profiles and a reduction of the computational burden of 2.13 and 43.90, respectively. Therefore, the line coding algorithm based on length and position of rows and columns shows the best performance among the assessed algorithms.

## 5. Conclusions

An innovative biometric system, which significantly improves the performance of previous systems developed by the research group, is presented in this paper. Its improvement has been achieved through an increment of the number of frequencies analyzed, a reduction of the number of scanning positions, the use of preprocessing techniques on acoustic images and the use of SVM algorithms for the classification task. Reliability and robustness of the system were improved by employing a large set of subjects called intruders, by increasing the number of acoustic profiles captured for each subject and by this regarding the clothes they were wearing during the test so as not to affect the classification.

It has been verified that, as the size of the acoustic profiles decreases by using the preprocessing techniques, the classification error increases, because relevant information is removed. However, the line coding algorithm based on length and position of rows and columns allows reducing computational burden in several orders of magnitude without increasing the classification error rate. In this case, the information of the profiles eliminated by the algorithm is not relevant to the classifier. Thus, this preprocessing algorithm has been selected to be used in the improved biometric system.

On the other hand, the fact that this line coding algorithm was based on binarized images shows that the relevant information for the classifier is associated to the contour of the image. Finally, it was observed that the geometric features extracted from the acoustic images do not provide enough information for the classifier.

Our research group is currently working on improving the biometric system by using bidimensional arrays, employing new algorithms based on Gaussian Mixture Models and creating a large database of acoustic profiles.
